# FSGT capsule inhibits IL‐1β‐induced inflammation in chondrocytes and ameliorates osteoarthritis by upregulating LncRNA PACER and downregulating COX2/PGE2

**DOI:** 10.1002/iid3.1334

**Published:** 2024-06-27

**Authors:** Mingyu He, Jian Liu, Yanqiu Sun, Xiaolu Chen, Jue Wang, Wu Gao

**Affiliations:** ^1^ Department of Rheumatism Immunity, The First Affiliated Hospital Anhui University of Chinese Medicine Hefei China; ^2^ Sinopharm Group Jingfang (Anhui) Pharmaceutical Co., Ltd. Jingfang China

**Keywords:** FSGTC, inflammation, LNCRNA, osteoarthritis, retrospective data mining

## Abstract

**Objective:**

To explore the efficacy and potential mechanism of Fengshi Gutong capsule (FSGTC) in osteoarthritis (OA) inflammation.

**Methods:**

The impact of FSGTC on laboratory indicators of OA patients was explored using data mining technology and association rule analysis. Then, the OA cell model was constructed by inducing chondrocytes (CHs) with interleukin‐1β (IL‐1β). In the presence of FSGTC intervention, the regulatory mechanism of PACER/COX2/PGE2 in OA‐CH viability and inflammatory responses was evaluated.

**Results:**

Retrospective data mining showed that FSGTC effectively reduced inflammation indexes (ESR, HCRP) of OA patients. Cell experiments showed that LncRNA PACER (PACER) silencing inhibited the proliferation activity of OA‐CHs, increased the level of COX2 protein, elevated the levels of PGE2, TNF‐α, and IL‐1β, and decreased the levels of IL‐4 and IL‐10 (*p* < .01). On the contrary, FSGTC‐containing serum reversed the effect of PACER silencing on OA‐CHs (*p* < .01). After the addition of COX2 pathway inhibitor, the proliferation activity of OA‐CHs was enhanced; the levels of PGE2, TNF‐α, and IL‐1β were decreased while the levels of IL‐4 and IL‐10 were increased (*p* < .01).

**Conclusion:**

FSGTC inhibits IL‐1β‐induced inflammation in CHs and ameliorates OA by upregulating PACER and downregulating COX2/PGE2.

## Graphical abstract

PACER is involved in the pathogenesis of osteoarthritis (OA) and can be used as a potential diagnostic marker of OA. Fengshi Gutong capsule (FSGTC) can inhibit the inflammatory response of OA by upregulating PACER and downregulating COX2/PGE2 expression. This study provides an important experimental basis and new direction for the treatment of OA by FSGTC.

## 1 INTRODUCTION

Osteoarthritis (OA), a chronic and degenerative joint disorder in the elderly population, is characterized by progressive cartilage destruction and subcartilage bone hyperplasia,^1^ causing joint pain and stiffness.^2^ The prevalence of OA is rising due to the growing aging of the population. Although OA affects both men and women, women are more likely to be symptomatic. OA occurs in 10% men and 18% in women aged 60 years or older.^3^, ^4^ OA has a multifactorial etiology, involving biomechanical factors, pro‐inflammatory mediators, and proteases.^5^ There is evidence to suggest that synovial inflammation is present in the OA joint and has been associated with joint tissue destruction and pain progression.^6^ Specifically, inflammation hydrolyzes extracellular matrix (ECM) by releasing cytokines such as interleukin‐1β (IL‐1β), nuclear factor‐κB (NF‐κB), tumor necrosis factor‐α (TNF‐α) to induce chondrocyte (CH) apoptosis and activate matrix metalloproteinase (MMP), eventually leading to articular cartilage degeneration.^7^, ^8^

Emerging studies have indicated the involvement of long noncoding RNA (lncRNA), a type of RNA molecule with over 200 nucleotides in length, in regulating OA inflammatory responses.^9^, ^10^ Ponting et al. found that considerable lncRNAs presented aberrant expression patterns during cartilage differentiation.^11^ Pearson et al. showed that lncRNA p50‐associated cyclooxygenase 2‐extragenic RNA (PACER) was downregulated in knee and hip OA cartilage.^12^ PACER is located upstream of the gene PTGS2 (COX‐2) and may affect OA inflammation by regulating COX‐2 expression.^13^ However, the specific regulatory mechanism of PACER in OA inflammation is still unclear. Therefore, this study aims to verify the expression of PACER by detecting serum samples from OA patients and further explores its regulatory mechanism in OA inflammation.

Due to the complexity of chronic diseases and the emergence of various comorbidities, the therapeutic effect of monotherapy on chronic complex diseases is relatively limited.^14^, ^15^ Accordingly, combination therapy has become a new trend in clinical research to improve efficacy and prevent drug resistance.^16^, ^17^ As a classic combination therapy, traditional Chinese medicine (TCM) has been used in the clinical practice for thousands of years.^18^, ^19^ TCM prescription, also known as formula, is composed of a variety of herbal ingredients with different pharmacological activities, which can produce synergistic effects in disease treatment.^20^, ^21^ Importantly, TCM compound has been reported to alleviate inflammatory responses in OA.^22^

Fengshi Gutong capsule (FSGTC) is a traditional herbal formula approved by the China Food and Drug Administration (No. Z34020025) and comprises seven herbs including ACONITI RADIX COCTA, ACONITI KUSNEZOFFII RADIX COCTA, CHAENOMELIS FRUCTUS, MUME FRUCTUS, CARTHAMI FLOS, EPHEDRAE HERBA, and GLYCYRRHIZAE RADIX ET RHIZOMA. Animal experiments^23^, ^24^ have shown that FSGTC can inhibit carrageenan‐caused paw tissue swelling and leukocyte migration in rats, reduce acetic acid‐caused writhing reactions in mice, improve inflammatory responses, and relieve pain in rats. A randomized controlled trial study has found that FSGTC can effectively improve the symptoms of OA patients, such as joint pain, poor flexion and extension, and walking difficulties, without adverse reactions.^25^ However, the specific mechanism of FSGTC improving OA inflammatory responses remains unclear. Therefore, we explored the improving effect of FSGTC on inflammatory indicators in OA patients through clinical data mining, determined the regulatory relationship between PACER and COX2 pathway, and verified the effect of FSGTC intervention through cell experiments, hoping to confer novel insights into the clinical treatment of OA.

## 2 MATERIALS AND METHODS

### 2.1 Clinical data of OA patients

The clinical data of OA patients hospitalized in the Rheumatology Department of the First Affiliated Hospital of Anhui University of Traditional Chinese Medicine from January 2012 to June 2013 were collected through the hospital information system. The collected data contained age, gender, and laboratory indicators, including erythrocyte sedimentation rate (ESR), hypersensitive C‐reactive protein (HCRP), total cholesterol (TC), triglyceride (TG), immunoglobulin A (IgA), immunoglobulin G (IgG), immunoglobulin M (IgM), and immune indicators (C3 and C4). After data screening and de‐duplication, the clinical data of 136 OA patients were finally obtained and used for retrospective analysis.

In addition,a total of 30 OA patients hospitalized from November 2021 to January 2022 were recruited, including six males and 24 females, with an average age of (61.43 ± 10.25) years. Additionally, 30 normal controls were recruited from the Physical Examination Center of the hospital, including five males and 25 females aged (61.32 ± 10.38) years. There was no statistically significant difference between the two groups at baseline (*p* > .05). Diagnostic criteria: all patients met the diagnostic criteria for OA proposed by the Chinese Rheumatology Society in 2010.^1^ Inclusion criteria: OA patients met the above western medical diagnosis. Exclusion criteria: (1) patients who did not meet the above diagnostic criteria; (2) lack of corresponding laboratory indicators after admission; (3) combined with other rheumatic diseases, such as systemic lupus erythematosus, ankylosing spondylitis, and so forth; (4) psychotic patients; (5) severe joint deformity and complete loss of joint function.

This study protected the privacy of the patients and did not interfere with the treatment plan. The Ethics Committee of the First Affiliated Hospital of Anhui University of Chinese Medicine waived the need for informed consent.

### 2.2 Association rule analysis

The Apriori module in IBM SPSS modeler 18.0 software was used to evaluate the correlation between FSGTC and the improvement of inflammatory indicators. The minimum support was defined as 15%, and the minimum confidence was defined as 50%.^26^

### 2.3 Preparation of FSGTC‐containing serum

FSGTC was purchased from Sinopharm Group Jingfang (Anhui) Pharmaceutical Co., Ltd. Twenty male Sprague‐Dawley (SD) rats were randomly assigned to the normal serum group and drug‐containing serum group. The rats in the normal serum group were given an equal amount of 0.9% normal saline, while the rats in the drug‐containing serum group were given FSGTC suspension (0.324 g/100 g) by tube feeding. The rats were given 2 mL/100 g by gavage once a day for 7 consecutive days. Two hours after the last administration, the rats were anesthetized with pentobarbital sodium (50 mg/kg), and the blood was collected from the abdominal aorta. Finally, FSGTC‐containing serum was obtained after centrifugation, inactivation, and sterilization of the blood. This study was supervised and approved by the Animal Ethics Committee of our hospital (AHUCM‐rats‐2022068).

### 2.4 CCK‐8 assay

Dulbecco's modified Eagle's medium (DMEM) (100 μL) containing 10% fetal bovine serum (FBS) was added into 96‐well plates (5 × 10^4^ cells/mL) for cell incubation at 37°C with 5% CO_2_ overnight. Then, all CHs (Human knee joint chondrocytes; iCell‐0092a; iCell Bioscience Inc.) were treated with the culture medium containing 10 μg/L IL‐1β^27^ for 24 h in each group and then treated with different concentrations of serum containing FSGT (5%, 10%, 15%, 20%, 25%, 30%) for 24, 48, and 72 h respectively, except the control group. Afterward, each well was supplemented with 10 μL CCK‐8 solution for another 1 h of incubation. Finally, the viability of CHs was evaluated by measuring the optical density value at 450 nm with enzyme‐linked immunosorbent assay (ELISA) (RT6100; China Leidu Life Sciences Co., Ltd.).

### 2.5 EdU assay

CHs in the logarithmic growth period were used for experiments. EdU solution was diluted with cell culture medium at the ratio of 1000:1 to prepare 50 mol/L EdU culture medium. CHs in each well were incubated with 100 μL 50 mol/L EdU culture medium for 2 h. Then, the culture medium was discarded, followed by phosphate‐buffered saline (PBS) (Servicebio, G4207, GP20060700991) rinsing three times (5 min/time). CHs in each well were treated with 100 μL cell fixative (i.e., PBS containing 4% paraformaldehyde) at room temperature for 30 min, and then, the fixative was discarded, followed by PBS rinsing three times (5 min/time). In the presence of 20 min of 0.5% Triton X‐100 (Servicebio, G1204, CR2,101,158) treatment and three times of PBS washing, CHs were stained with 2 mg/mL glycine in a decolorized shaker for 5 min. Following PBS washing three times, CHs in each well were treated with 100 μL Appollo dye reaction solution in a decolorization shaker devoid of light for 30 min. Next, 100 μL methanol was added into each well for cleaning twice (5 min/time). CHs were incubated with Hoechst 33342 staining solution diluted with PBS buffer at a ratio of 1000:1 in the dark for 5 min and washed with PBS buffer three times (5 min/time. Finally, an inverted fluorescence microscope (OLYMPUS, CKX53) was used for image capture.

### 2.6 Quantitative reverse‐transcription polymerase chain reaction (qRT‐PCR)

The total RNA was extracted from OA‐CHs using TRIzol reagents. The relative expression of genes was analyzed using the $2^{‐\Delta \Delta C_{t}}$ method,^28^ with β‐actin as the internal reference.^29^, ^30^ The upstream primer of β‐actin is 5′‐CCCTGGAGAAGAGCTACGAG‐3′, and the downstream primer is 5′‐GGAGAGGAGAAGGCTGAGAGT‐3′; the product length is 96 bp. The upstream primer of PACER is 5′‐TCTTTTCGCAGTCTTGCC‐3′, and the downstream primer is 5′‐GAAGCCAAGTGTCCTTCTGC‐3′; the product length is 98 bp.

### 2.7 ELISA

The contents of PGE2, TNF‐α, IL‐1β, IL‐4, and IL‐10 in the culture supernatant of CHs were detected using the corresponding ELISA kits (RX105367H, RX104793H, RX106152H, RX106128H, and RX103064H; Quanzhou Ruixin Biotechnology Co., Ltd.).

### 2.8 Western blot

The cell precipitation was collected into six‐well plates. Each well was added with 100 μL radioimmuno‐precipitation assay (RIPA) lysate (BL504A; Biosharp) to lyse the CHs and collect the supernatant. After the collected protein was added into the loading well in a ratio of 1:4, it was separated by sodium dodecyl sulfate‐polyacrylamide gel electrophoresis for 1 h and then transferred onto the polyvinylidene fluoride membrane. After that, the protein membrane was immediately placed in the prepared Western washing solution to wash away the membrane transfer solution. Then, the membrane was blocked with 5% skim milk powder (note: 5% bovine serum albumin [BSA] was selected for phosphorylated protein blocking) at room temperature for 2 h, incubated overnight with the primary antibodies (β‐actin, goat anti‐mouse IgG, goat anti‐rabbit IgG, and COX2), and then incubated with the horseradish peroxidase‐labeled secondary antibody (1:10000) at room temperature. Finally, electrochemiluminescence hypersensitive luminescence kit was used to measure protein. The absorbance value of the target protein was calculated with β‐actin as the internal control, and Image J software was used for protein band analysis.

### 2.9 Statistical analysis

SPSS 23.0 (IBM) and GraphPad Prism 8.0 were used for data analysis and plotting, respectively. The laboratory indicators of patients before and after treatment were analyzed by Wilcoxon's signed‐rank test and expressed in the median (quartile range). *p* < .05 was considered to indicate a statistical difference.

## 3 RESULTS

### 3.1 Effect of FSGTC on clinical laboratory indicators of OA patients

The levels of ESR, HCRP, TC, and TG were significantly reduced after FSGTC treatment (*p* < .01), while there was no significant difference in the changes of IgA, IgG, IgM, C3, and C4 before and after FSGTC treatment (*p* > .05) (Table S1). Association rule analysis manifested that FSGTC improved ESR [94.85% confidence interval (CI)], HCRP (95.59% CI), TC (66.74% CI), and TG (62.09% CI) (Figure S1). These results suggested FSGTC was closely correlated to the improvement of inflammatory markers in OA patients.

### 3.2 Expressions of PACER/COX2/PGE2 and inflammatory factors in OA patients

**Figure 1:**
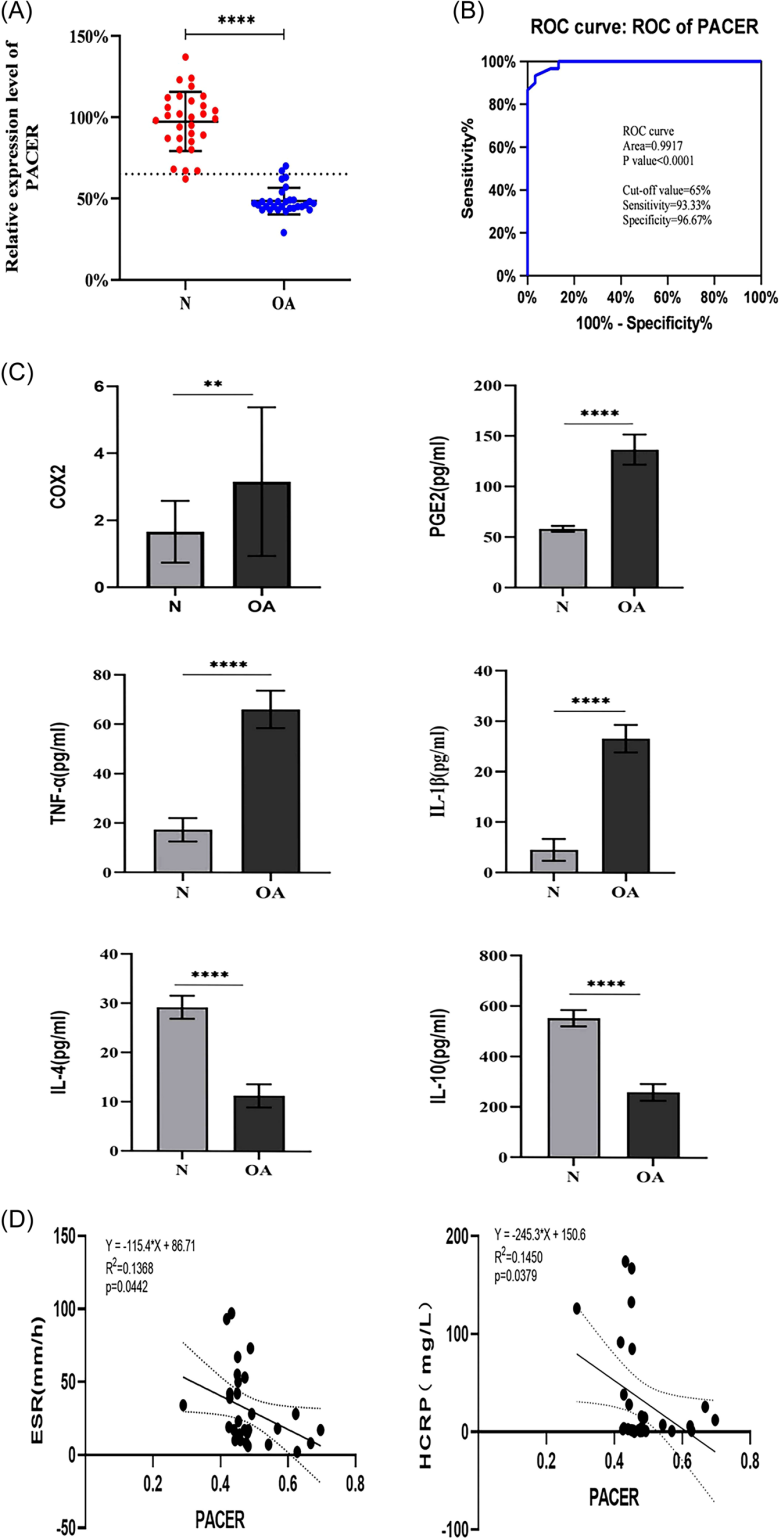
Expression of PACER/COX2/PGE2 and inflammatory factors in OA patients. (A) Change of PACER in OA patients. *N* = 30, unpaired Student's *t* test; (B) ROC curve analysis; (C) expression of COX2/PGE2 and inflammatory factors (TNF‐α, IL‐1β, IL‐4, IL‐10) in serum of patients with OA, *N* = 30, unpaired Student's *t* test; (D) *N* = 30, Spearman's correlation analysis. IL‐1β, interleukin‐1β; OA, osteoarthritis; PACER, p50‐associated cyclooxygenase 2‐extragenic RNA; ROC, receiver operating characteristic; TNF‐α, tumor necrosis factor‐α. ****p < .0001; **p < .01.

The peripheral blood samples of 30 normal controls and 30 OA patients were subjected to qRT‐PCR and ELISA assays. The results showed that the expression of PACER in OA patients was significantly reduced compared with that in the normal controls (*p* < .05, Figure 1A). The area under the receiver operating characteristic (ROC) curve of PACER was 0.9917 (Figure 1B), indicating the diagnostic value of PACER. Meanwhile, the levels of COX2, PGE2, and pro‐inflammatory factors (TNF‐α and IL‐1β) were increased, but the levels of IL‐4 and IL‐10 were decreased (Figure 1C). Spearman correlation analysis showed that PACER was negatively correlated with ESR, HCRP, and D‐D (*p* < .05, Figure 1D). These results indicated that the elevation of inflammatory indicators in OA patients was related to the decrease of PACER and the activation of COX2/PGE2.

### 3.3 Expressions of PACER/COX2/PGE2 and inflammatory factors in OA‐CHs induced by IL1‐β

**Figure 2:**
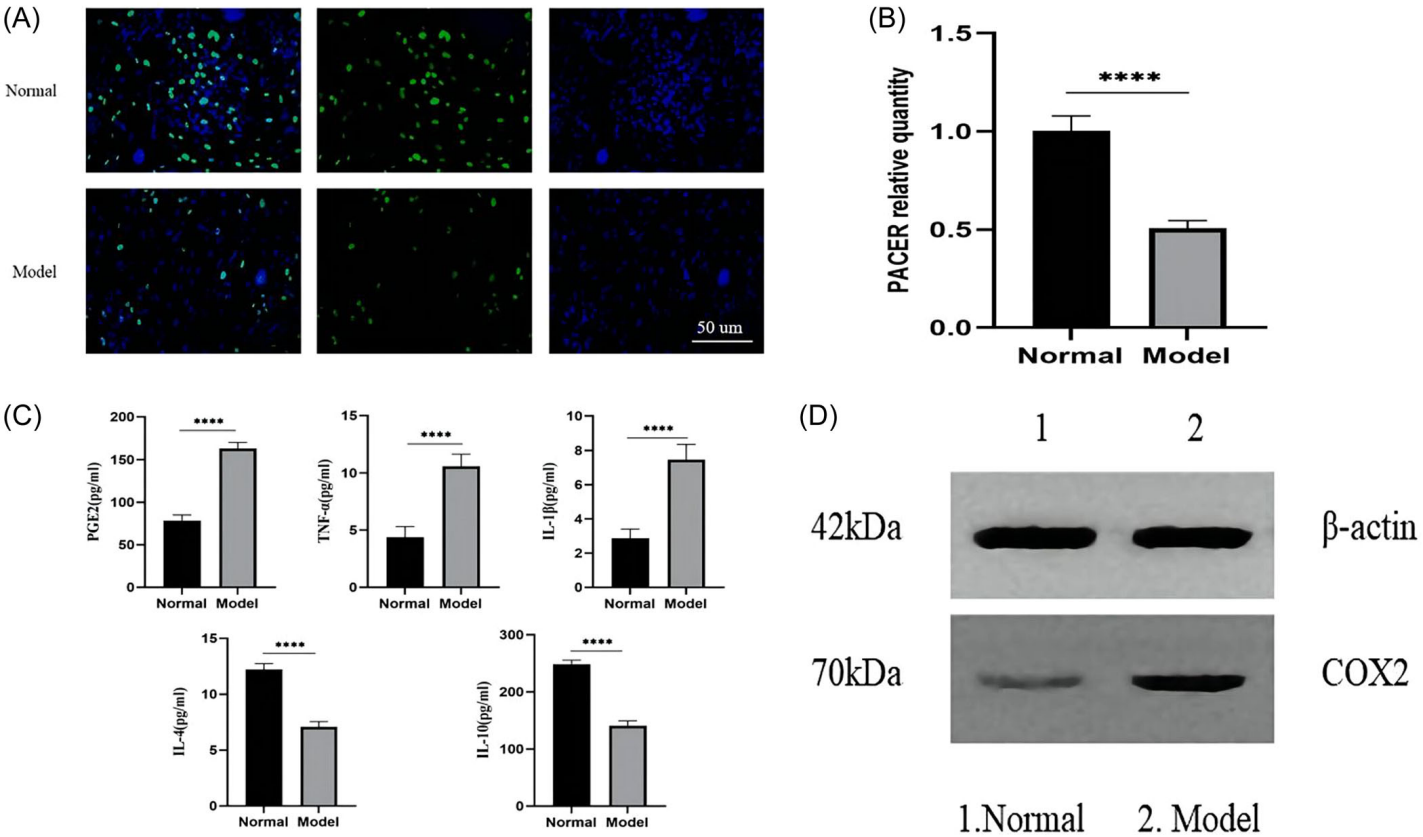
Expression of PACER/COX2/PGE2 and inflammatory factors in IL1‐β + OA‐CHs. (A) Proliferation activity of CHs in IL1‐β + OA‐CHs; (B) expression level of PACER in IL1‐β + OA‐CHs; (C) expression level of PGE2 and inflammatory factors (TNF‐α, IL‐1β, IL‐4, IL‐10) in IL1‐β + OA‐CHs; (D) COX2 protein level in IL1‐β + OA‐CHs. Scale bar: 200 µm. *N* = 8, unpaired Student's *t* test. CH, chondrocyte; IL‐1β, interleukin‐1β; OA, osteoarthritis; PACER, p50‐associated cyclooxygenase 2‐extragenic RNA; TNF‐α, tumor necrosis factor‐α. ****p < .0001.

EdU staining showed that compared with control CHs, OA‐CHs induced by IL1‐β had inhibited proliferation activity (Figure 2A), reduced PACER expression (*p* < .01, Figure 2B), and increased COX2 protein level (Figure 2D). Moreover, the levels of PGE2, TNF‐α, and IL‐1β were increased, while the levels of IL‐4 and IL‐10 were decreased in OA‐CHs (*p* < .01, Figure 2C). Based on this model, we conducted further research.

### 3.4 Effect of PACER overexpression or silencing on OA‐CHs, COX2/PGE2, and inflammatory factors

**Figure 3:**
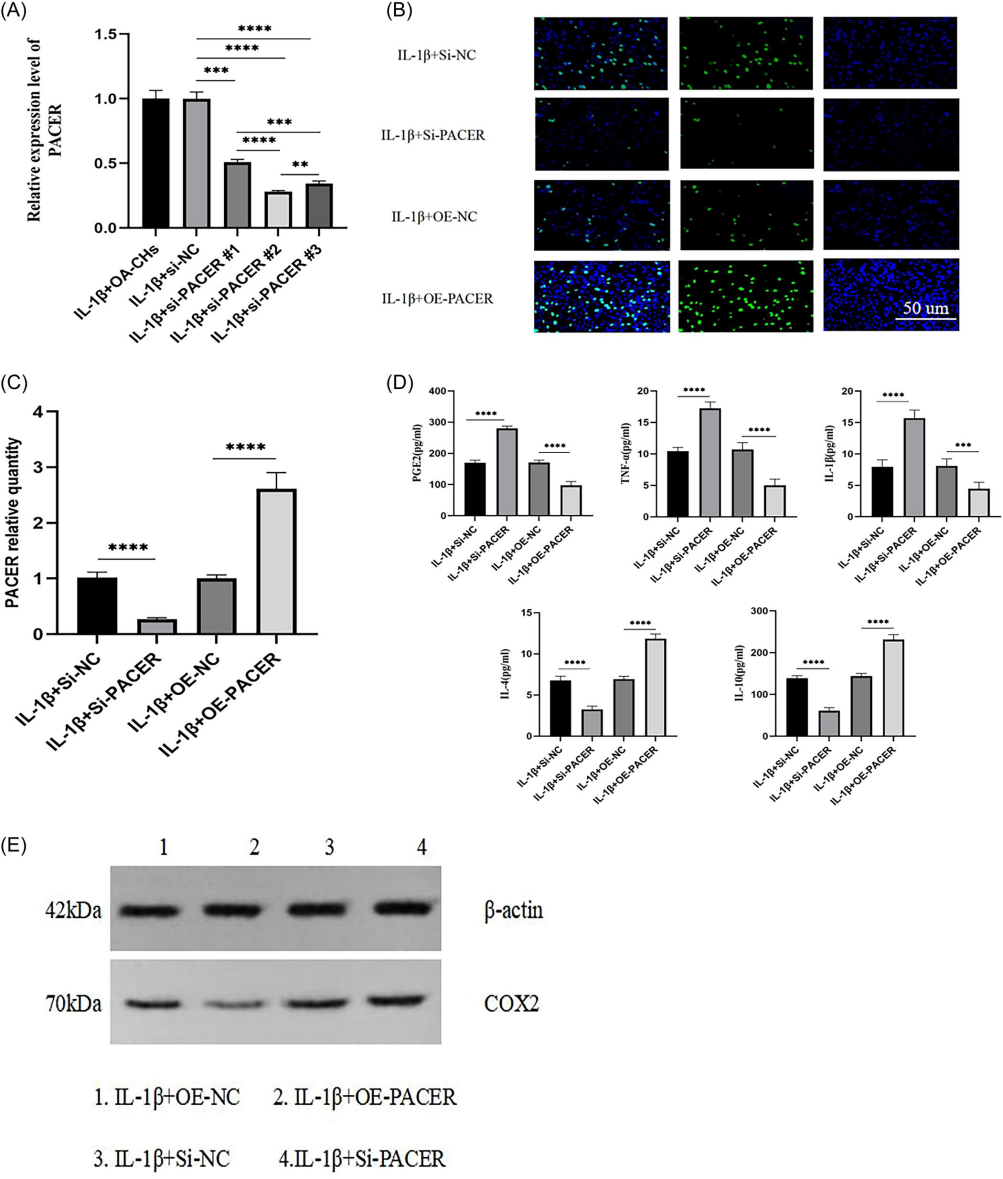
Effects of overexpression or silencing of PACER on OA‐CHs function, COX2/PGE2 and inflammatory factors. (A, C) qRT‐PCR is used to determine the level of PACER; (B) EDU was used to determine the cell viability of OA‐CHs in PACER overexpression or silencing state; (D) ELISA is used to evaluate the levels of PGE2, TNF‐ α, IL‐1 β, IL‐4 and IL‐10; (E) WB was used to determine the expression of COX2 protein. Scale bar: 200 µm. *N* = 8, unpaired Student's *t* test. CH, chondrocyte; ELISA, enzyme‐linked immunosorbent assay; IL‐1β, interleukin‐1β; OA, osteoarthritis; PACER, p50‐associated cyclooxygenase 2‐extragenic RNA; qRT‐PCR, quantitative reverse‐transcription polymerase chain reaction; TNF‐α, tumor necrosis factor‐α. ****p < .0001; ***p < .001; **p < .01.

Subsequently, we constructed the siRNA targeting PACER, and the expression of PACER in OA‐CHs was significantly downregulated after transfection of si‐PACER#2, so we chose the si‐PACER#2 for subsequent experiments (Figure 3A). To further verify the regulatory effect of PACER on OA, we edited PACER (Figure 3C) to observe the effect of PACER overexpression or silencing on OA‐CHs, COX2/PGE2, and inflammatory factors. The results showed that the proliferation activity of OA‐CHs transfected with si‐PACER was significantly inhibited (Figure 3B), and the COX2 protein level was increased (Figure 3E); in addition, the levels of PGE2, TNF‐α, and IL‐1β were increased, while the levels of IL‐4 and IL‐10 were decreased after PACER silencing (*p* < .01, Figure 3D). PACER overexpression led to the opposite results (Figures 3B,E,D).

### 3.5 PACER regulated OA‐CH viability and inflammatory responses via COX2/PGE2

**Figure 4:**
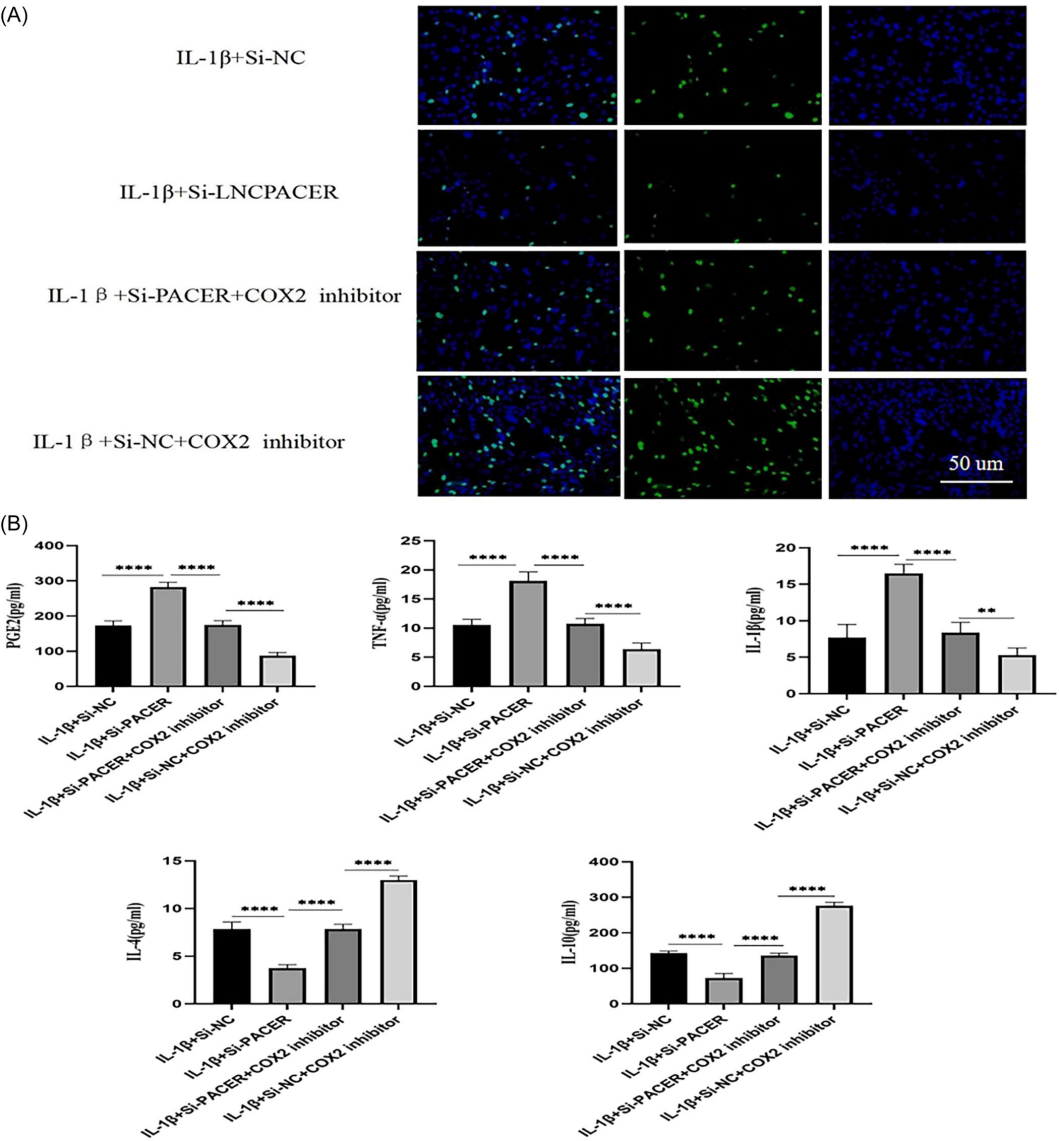
PACER regulates the activity of OA‐CHs and the expression of inflammatory factors through COX2/PGE2; (A) The OA‐CHs proliferation activity at PACER interference status and COX2 inhibition status; (B) the expression level of PGE2 and inflammatory factors at PACER interference status and COX2 inhibition status. Scale bar: 200 µm. *N* = 8, unpaired Student's *t* test. CH, chondrocyte; IL‐1β, interleukin‐1β; OA, osteoarthritis; PACER, p50‐associated cyclooxygenase 2‐extragenic RNA. ****p < .0001; **p < .01.

Compared with those in the IL‐1β + si‐NC group, OA‐CHs in the IL‐1β + si‐PACER group showed inhibited proliferation activity (Figure 4A); increased levels of PGE2, TNF‐α, and IL‐1β, decreased levels of IL‐4 and IL‐10 (*p* < .01, Figure 4B). However, the addition of COX2 pathway inhibitor enhanced the proliferation activity of OA‐CHs (Figure 4A), decreased the levels of PGE2, TNF‐α, and IL‐1β, and increased the levels of IL‐4 and IL‐10 (*p* < .01, Figure 4B). It was indicated that PACER silencing augmented the release of PGE2, inhibited the proliferation of OA‐CHs, and promoted inflammation by activating COX2.

### 3.6 FSGTC reversed the effect of PACER silencing on OA‐CHs

**Figure 5:**
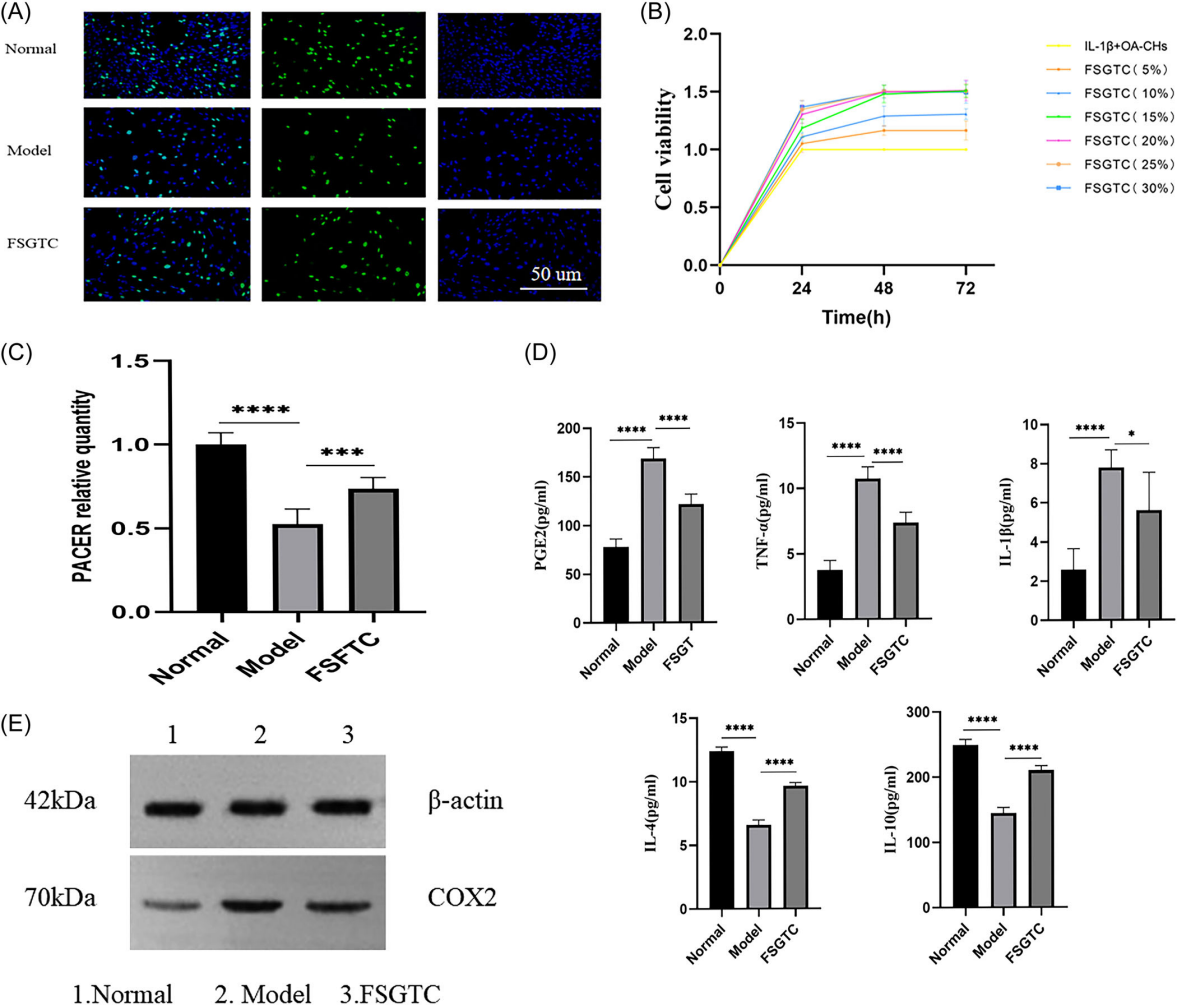
FSGTC‐containing serum reverses the effect of silenced LNCC0068 on OA‐CHs inflammation. (A,B) EDU and CCK‐8 are used to determine the cell viability of OA‐CHs interfered by FSGTC‐containing serum. (C) qRT‐PCR is used to determine the level of PACER. (D) ELISA is used to evaluate the levels of PGE2 and TNF‐ α, IL‐1 β, IL‐4, and IL‐10. (E) WB was used to determine the expression of COX2 protein. Scale bar: 200 µm. *N* = 8, unpaired Student's *t* test. CH, chondrocyte; FSGTC, Fengshi Gutong capsule; IL‐1β, interleukin‐1β; OA, osteoarthritis; qRT‐PCR, quantitative reverse‐transcription polymerase chain reaction; TNF‐α, tumor necrosis factor‐α. ****p < .0001; ***p < .001; *p < .05.

Previous studies have reported the role of PACER in inflammatory responses of OA‐CHs.^13^ Further, we hypothesized that FSGTC might affect the inflammatory response of OA by regulating the expression of PACER. To verify it, we evaluated the effect of FSGTC‐containing serum on the proliferation activity of OA‐CHs induced by IL1‐β (Figure 5A,B). The cell proliferation activity in the model group was significantly inhibited compared with that in the control group. With the increase of FSGTC‐containing serum concentration, the proliferation activity of OA‐CHs was enhanced. After treatment with different concentrations of FSGTC‐containing serum, the proliferation activity of OA‐CHs at 48 and 72 h was weakened compared to 24 h. However, there was no significant difference in the proliferation activity of OA‐CHs between 48 and 72 h, so 48 h was chosen as the optimal intervention time. At 48 h, there were significant differences in the proliferation activity of OA‐CHs between the 15%, 20%, 25%, and 30% groups with the 5% and 10% groups (*p* < .05), while there was no significant difference between the 15%, 20%, 25%, and 30% groups. Therefore, 15% FSGTC‐containing serum was finally selected for the following experiments. The effect of FSGTC‐containing serum on PACER, COX2/PGE2, and inflammatory cytokines were evaluated using qRT‐PCR, WB, and ELISA. The results showed that FSGTC‐containing serum increased the levels of PACER, IL‐4, and IL‐10, but decreased the levels of COX2, PGE2, TNF‐α, and IL‐1β (Figure 5C–E).

### 3.7 FSGTC reversed the effect of PACER silencing on COX2/PGE2 in OA‐CHs

**Figure 6:**
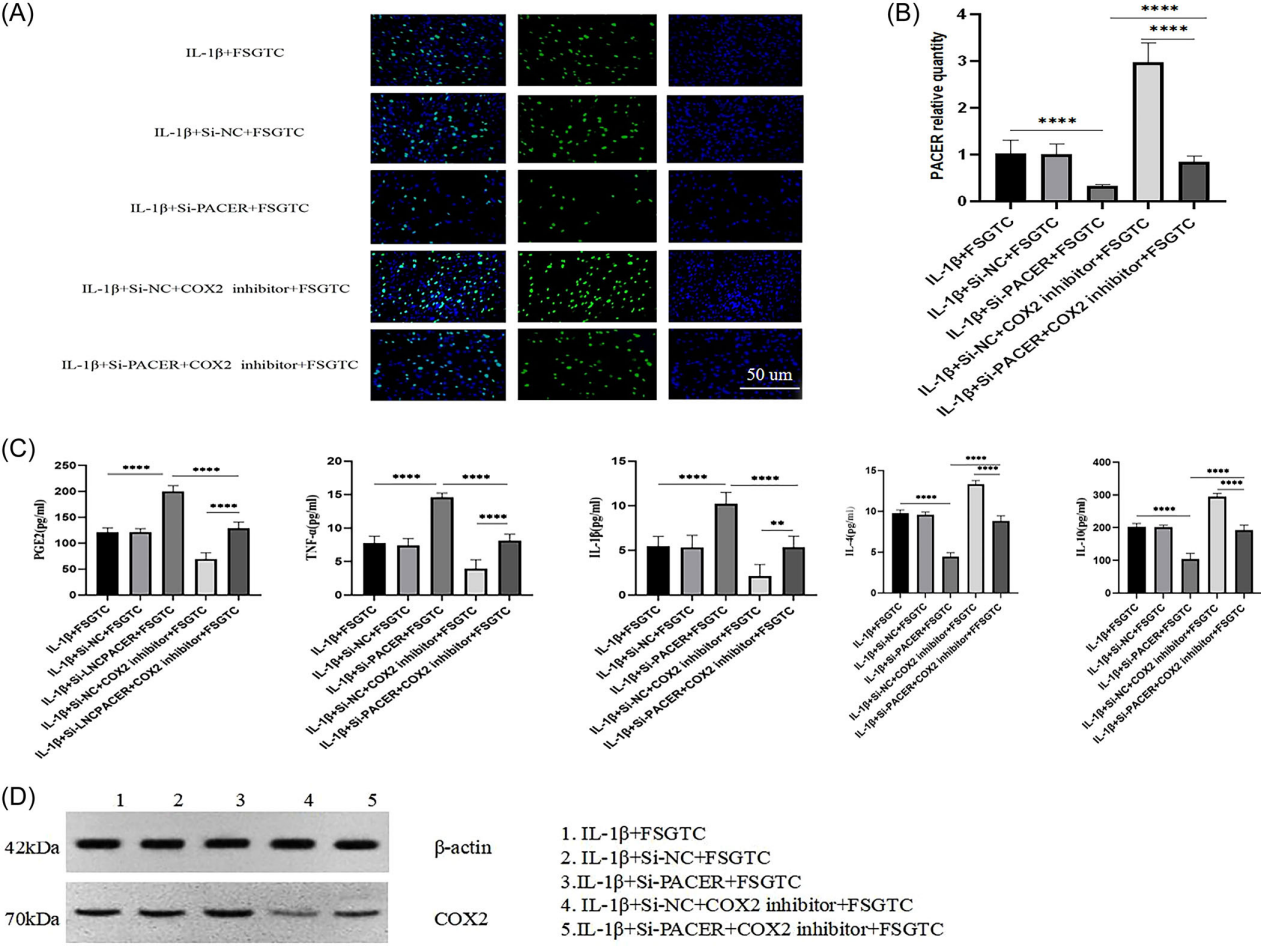
FSGTC‐containing serum reverses the effect of si‐PACER on COX2/PGE2 in OA‐CHs. (A) EDU was used to determine the cell viability of OA CHs; (B) qRT‐PCR was used to determine the level of PACER; (C) ELISA is used to evaluate the levels of PGE2 and TNF‐ α, IL‐1 β, IL‐4, and IL‐10; (D) Western blot for determination of COX2 protein expression. Scale bar: 200 µm. *N* = 8, unpaired Student's *t* test. CH, chondrocyte; FSGTC, Fengshi Gutong capsule; IL‐1β, interleukin‐1β; OA, osteoarthritis; PACER, p50‐associated cyclooxygenase 2‐extragenic RNA; qRT‐PCR, quantitative reverse‐transcription polymerase chain reaction; TNF‐α, tumor necrosis factor‐α. ****p < .0001, **p < .01.

As shown in Figure 6, we conducted rescue experiments to determine whether the effect of FSGTC‐containing serum on OA‐CHs was achieved by regulating the expression of PACER. The results showed that FSGTC offset the effect of si‐PACER treatment on the levels of COX2, PGE2, TNF‐α, and IL‐1β in OA‐CHs to some extent, partially restored the proliferation activity of OA‐CHs, and increased the levels of IL‐4 and IL‐10 (*p* < .01, Figure 6A–D). Finally, rescue experiments were performed to determine whether PACER participated in the regulation of FSGTC on COX2. The results showed that FSGTC treatment partially reversed the level of COX2 protein in OA‐CHs in the si‐PACER group. The effect of FSGTC on the COX2 pathway regulated by PACER was further verified in OA‐CHs. The results showed that the addition of COX2 pathway inhibitor, to some extent, offset the levels of PGE2, TNF‐α, and IL‐1β in OA‐CHs transfected with si‐PACER, and partially restored the proliferation activity of OA‐CHs and the levels of IL‐4 and IL‐10 (*p* < .01, Figure 6A–D).

## 4 DISCUSSION

OA has traditionally been defined as a noninflammatory arthropathy. However, emerging evidence further refines the disease interpretation and indicates the true inflammatory characteristics of OA.^31^ A variety of inflammatory mediators including IL‐1β, IL‐6, and TNF‐α can be detected in the synovial fluid and serum of patients with OA, indicating that OA is not a local noninflammatory disease. These inflammatory mediators drive the occurrence and development of OA, causing articular cartilage damage and synovial inflammation.^32^, ^33^ Although drug therapy has the potential to alleviate the inflammatory reaction of OA patients and delay the disease progression, there is no proven disease modifier for OA yet.^34^ TCM is characterized by multi‐components, multi‐targets, and multi‐channels, which has been accepted as a complementary therapy for OA.^35^ For instance, TCM is reported to relieve the inflammatory response of OA patients by regulating the key proteins of immune inflammation.^36^, ^37^ This study used retrospective data mining to analyze the clinical data of 136 OA patients treated with FSGTC and clarified that FSGTC effectively improved the inflammatory reaction and lipid metabolism disorder of OA patients. Subsequent association rule analysis found that FSGTC improved HCRP, ESR, TC, and TG, with a confidence level of 94.85%, 95.59%, 66.74%, and 62.09%, respectively, indicating that FSGTC was closely related to the improvement of inflammation and lipid metabolism markers in OA patients.

Accumulating studies have shown that dysregulated lncRNAs contribute to the pathogenesis of OA by influencing CH proliferation, inflammatory responses, and angiogenesis.^38^, ^39^ COX‐2, an enzyme that catalyzes arachidonic acid to produce prostaglandins, can be induced by cytokines and inflammatory signals to participate in various physiological and pathological processes such as inflammation and pain.^40^ LncRNA PACER has been demonstrated to promote the expression of COX‐2 by binding to the inhibitory transcription factor p50.^13^ Through small sample clinical verification, this study found that PACER expression was downregulated in the serum of OA patients and was negatively correlated with laboratory indicators including ESR, HCRP, and D‐D (*p* < .05). Moreover, the expressions of COX2, PGE2, TNF‐α, and IL‐1β were increased, while the expressions of IL‐4 and IL‐10 were decreased, indicating that the presence of inflammatory responses in OA patients was related to the decrease of PACER and the activation of COX2/PGE2.

To further explore the regulatory relationship between PACER and COX2 pathway, we constructed an OA‐CH model by IL1‐β induction to observe the effect of PACER overexpression or silencing on OA‐CHs, COX2/PGE2, and inflammatory factors. IL‐1β, as a key inflammatory cytokine in the pathological progression of OA, can mediate the production of other inflammatory factors such as PGE2, IL‐6, and so forth, ultimately leading to joint dysfunction.^41^, ^42^ Therefore, IL‐1β is often used to establish in vitro models of OA. Accordingly, this study induced CHs by IL‐1β to simulate the inflammatory internal environment of OA.

The results showed that PACER silencing inhibited the proliferation activity of OA‐CHs, increased the level of COX2 protein, elevated the levels of PGE2, TNF‐α, and IL‐1β, decreased the levels IL‐4 and IL‐10 (*p* < .01). PACER overexpression led to the opposite trends. The addition of COX2 pathway inhibitor enhanced the proliferation activity of OA‐CHs, decreased the levels of PGE2, TNF‐α, and IL‐1β, and increased the levels IL‐4 and IL‐10 (*p* < .01). Briefly, PACER was poorly expressed in OA patients, which promoted the release of PGE2 by activating COX2, inhibited the proliferation of CHs, and promoted inflammation.

FSGTC created by China National Medicine Jingfang Group has been used in clinical practice for more than 20 years and exhibited significant therapeutic effects. Systematic research has been conducted on FSGTC in the treatment of OA from theory, pharmacy, clinical, and experimental aspects. For example, Chen et al. showed that FSGTC combined with Voltalin tablets could increase the therapeutic efficacy and reduce the recurrence rate of OA without additional adverse reactions compared with the use of FSGTC or Voltalin tablets alone.^43^ Jia et al., found that the combination of FSGTC and rehabilitation training program had a significant effect for patients with early knee OA.^44^ Our study showed that FSGTC upregulated the expression of PACER in OA‐CHs, downregulated the expressions of COX2/PGE2, reduced the levels of pro‐inflammatory factors, and elevated anti‐inflammatory factors, thereby improving OA inflammation and alleviating pain, swelling, and other symptoms of OA patients.

The curative effect of FSGTC in clinical application is definite, but the aconitine alkaloids prepared from ACONITI RADIX COCTA and ACONITI KUSNEZOFFII RADIX COCTA in this prescription are both effective and toxic components, with cardiovascular toxicity, neurotoxicity, and digestive system toxicity.^45^, ^46^ *Pharmacopoeia of the People's Republic of China (2020 Edition): Volume I* contains the quality standard of FSGTC.^47^ This standard has identified EPHEDRAE HERBA by thin layer chromatography (TLC), checked the limit of aconitine by TLC and determined the content of total alkaloids and ephedrine in aconitum by ultraviolet spectrophotometry and high‐performance liquid chromatography. Chen et al. improved and optimized the quality standard of FSGTC on the basis of the original standard so as to control the quality of FSGTC more systematically and provide a scientific basis and reference for the safe use of FSGTC in the clinic.^48^ Peng et al. also found that FSGTC only had a mild toxic reaction to the heart when it was 60 times the clinical dosage, indicating the safety of FSGTC in the clinical application.^49^ In this study, the CCK‐8 assay showed that serum containing FSGTC at different concentrations increased the proliferation activity of OA‐CHs induced by IL‐1β. In addition, EdU staining showed that FSGTC treatment partially restored the proliferation activity of OA‐CHs transfected with si‐PACER.

## 5 STUDY LIMITATION

The current study also has some limitations. For example, The clinical data included in this study were limited to the First Affiliated Hospital of Anhui University of Chinese Medicine, and our data failed to be updated in a timely manner. In the later stage, the sample size should be expanded, and multicenter clinical data mining research should be conducted. Then, since FSGTC is multi‐component and multi‐target in the treatment of OA, this study is a preliminary exploration of the complex mechanism of FSGTC. Further cell experiments are warranted to excavate the complex mechanism of FSGTC in treating OA.

## 6 CONCLUSION

PACER is involved in the pathogenesis of OA and can be used as a potential diagnostic marker of OA. FSGTC can inhibit the inflammatory response of OA by upregulating PACER and downregulating COX2/PGE2. This study provides an important experimental basis and new direction for the treatment of OA by FSGTC.

## AUTHOR CONTRIBUTIONS

Jue Wang, Wu Gao, and Jian Liu contributed to the conception and design of this study. Mingyu He, Yanqiu Sun, and Xiaolu Chen were responsible for the experimental data collection and analysis. All authors read and approved the final version of the manuscript. Mingyu He was responsible for writing the manuscript.

## CONFLICT OF INTEREST STATEMENT

The authors declare no conflict of interest.

## ETHICS STATEMENT

Our experiment was supervised by the Ethics Committee for Laboratory Animals of the First Affiliated Hospital of Anhui University of Traditional Chinese Medicine approved this study (approval no. AHUCM‐rats‐2022068).

## Supporting information

Supporting information.

Supporting information.

## Data Availability

The data sets used and/or analyzed during the current study are available from the corresponding author on reasonable request.
