# Supplementary figures and images for: FSGT capsule inhibits IL‐1β‐induced inflammation in chondrocytes and ameliorates osteoarthritis by upregulating LncRNA PACER and downregulating COX2/PGE2
Source: Immun Inflamm Dis. 2024 Jun 27;12(6):e1334. doi: 10.1002/iid3.1334 (PMC11211208; doi:10.1002/iid3.1334)

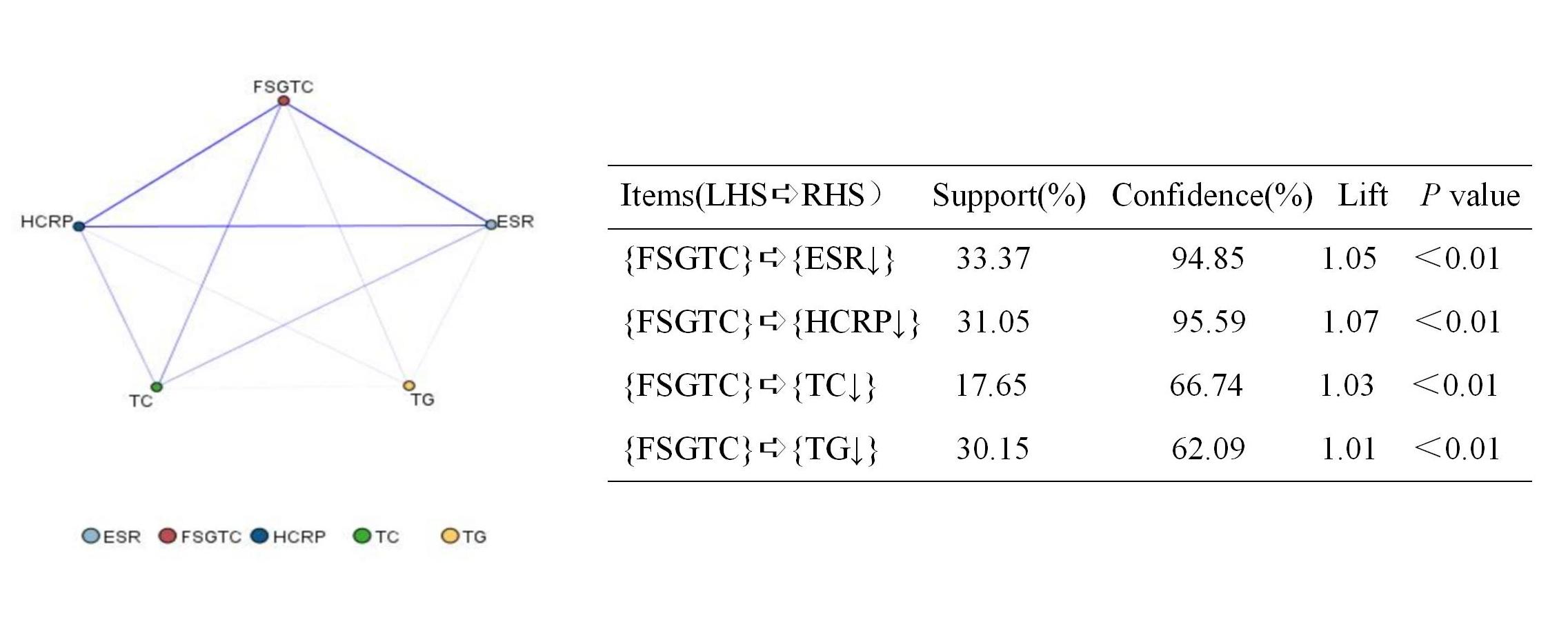

Supplement: Supplementary file 1 — Supporting information. [file IID3-12-e1334-s002.jpg]
