# Supplementary material for: FSGT capsule inhibits IL‐1β‐induced inflammation in chondrocytes and ameliorates osteoarthritis by upregulating LncRNA PACER and downregulating COX2/PGE2
Source: Immun Inflamm Dis. 2024 Jun 27;12(6):e1334. doi: 10.1002/iid3.1334 (PMC11211208; doi:10.1002/iid3.1334)
Supplement: Supplementary file 2 — Supporting information. [file IID3-12-e1334-s001.docx]

supplementary Table S1 Effect of FSGTC on clinical laboratory indexes of OA patients.

Variables Before treatment After treatment P value

ESR (mm/h) 25.471(16.000,29.750) 13.912(8.000,17.000) ＜0.01

HCRP (mg/L) 12.784(7.723,13.200) 4.401(4.373,5.457) ＜0.01

TC (mmol/L) 4.767(3.943,5.333) 4.672(3.868,5.328) ＜0.01

TG (mmol/L) 1.864(1.213,2.480) 1.665(0.953,2.283) 0.030

IgA (g/L) 2.159(1.558,2.633) 2.155(1.558,2.633) 0.075

IgG (g/L) 11.512(9.900,13.050) 11.482(9.900,13.050) 0.054

IgM (g/L) 1.127(0.780,1.463) 1.127(0.780,1.470) 0.169

C3 (g/L) 105.828(91.950,118.875) 105.352(91.600,117.825) 0.331

C4 (g/L) 25.023(19.725,28.775) 24.943(19.825,28.775) 0.133
